# Mechanical Properties of Nano-SiO_2_ Reinforced Geopolymer Concrete under the Coupling Effect of a Wet–Thermal and Chloride Salt Environment

**DOI:** 10.3390/polym14112298

**Published:** 2022-06-05

**Authors:** Qingqing Jin, Peng Zhang, Jingjiang Wu, Dehao Sha

**Affiliations:** 1 School of Water Conservancy Engineering, Zhengzhou University, Zhengzhou 450001, China; jinqingqing1020@163.com (Q.J.); a18637195898@163.com (D.S.); 2 Communications Construction Company of CSCEC 7th Division Co., Ltd., Zhengzhou 450004, China; wujingjiang@cscec.com

**Keywords:** geopolymer concrete (GPC), nano-SiO_2_ (NS), mechanical properties, wet–thermal and chloride salt environment

## Abstract

In this study, the mechanical behaviors of nano-SiO_2_ reinforced geopolymer concrete (NS-GPC) under the coupling effect of a wet–thermal and chloride salt environment were investigated through a series of basic experiments, and a simulation on the coupling effect of a wet–thermal and chloride salt environment and SEM test were also included. During the experiments for the coupling effect of the wet–thermal and chloride salt environment, an environment simulation test chamber was utilized to simulate the wet–thermal and chloride salt environment, in which the parameters of relative humidity, temperature, mass fraction of NaCl solution and action time were set as 100%, 45 °C, 5% and 60 d, respectively. The content of nano-SiO_2_ (NS) particles added in geopolymer concrete (GPC) were 0, 0.5%, 1.0%, 1.5% and 2.0%. The result indicated that the mechanical properties of NS reinforced GPC decreased under the coupling effect of the wet–thermal and chloride salt environment compared to the control group in the natural environment. When the NS content was 1.5%, the cube and splitting tensile strength, elastic modulus and impact toughness of GPC under the coupling environment of wet–thermal and chloride salt were decreased by 9.7%, 9.8%, 19.2% and 44.4%, respectively, relative to that of the GPC under the natural environment. The addition of NS improved the mechanical properties of GPC under the coupling effect of the wet–thermal and chloride salt environment. Compared to the control group without NS, the maximum increment in cube compressive strength, splitting tensile strength and elastic modulus of NS–GPC under the coupling effect of the wet–thermal and chloride salt environment due to the incorporation of NS reached 25.8%, 9.6% and 17.2%, respectively. Specifically, 1.5% content of NS increased the impact toughness, impact numbers of initial crack and the ultimate failure of GPC by 122.3%, 109% and 109.5%, respectively.

## 1. Introduction

Ordinary Portland cement (OPC) is the most commonly applied building material because of its favorable mechanical behaviors and durability, good bonding with steel and low cost; however, it also has insufficient toughness, low tensile strength, brittleness and other initial defects, which often leads to structural failure or even unserviceability in practical engineering [1]. Besides, with the growing demand for concrete, a large amount of OPC is produced, requiring considerable resource and energy consumption, resulting in massive CO_2_ emissions [2]. The widespread application of OPC is believed to be one of the primary causes of global warming, so it is necessary to find other more environmentally friendly building materials to replace the OPC. Davidovits first proposed geopolymers, which were prepared by activating active aluminosilicate materials in a highly alkaline solution [3]. Geopolymers have the characteristics of fast hardening, early strength, excellent mechanical behaviors, good corrosion and high temperature resistance, and they can provide greater environmental benefits than those of OPC [4,5]. Low-cost raw materials from industrial waste and geological sources can be used in the preparation of geopolymers, including metakaolin (MK), fly ash (FA), slag, bottom ash, etc. [6,7]. Compared to the preparation technology of geopolymer requiring high-temperature curing and single raw materials in the past, geopolymer has gradually developed from traditional materials such as MK to industrial waste materials such as FA in recent years [8,9,10]. Several researchers have illustrated that the synergistic effect of MK and FA grants geopolymers excellent mechanical behaviors and high temperature resistance [11,12,13,14,15].

Geopolymer concrete (GPC) has the advantages of environmental protection and energy savings, as well as mechanical properties close to or better than OPC concrete (OPCC) [16,17,18,19,20]. Besides, GPC will show higher temperature resistance on some structures, compared to OPCC [21]. Hassan et al. [22] discovered that GPC had slight dry shrinkage and needed a shorter curing time than OPCC. Moreover, the durability of GPC has an important influence on its performance [23,24]. Bakri et al. [25] also studied that FA-based GPC had superior acid resistance relative to OPCC. However, the mechanical properties and durability of traditional GPC cannot fully meet the requirements in the engineering in a severe environment [21]. Therefore, some researchers introduced different kinds of materials to further enhance the durability and toughness of GPC, such as nanomaterials, polyvinyl alcohol fibers, steel fibers and so on [26,27,28,29]. Adding nanomaterial into the matrix is one of the most effective methods for enhancing the applicability and improving the mechanical properties and durability of GPC.

Due to the superfine size, large specific surface and high reactivity of nanoparticles, the addition of nanomaterials can greatly enhance the mechanical performances and durability of GPC. Some academics have carried out much research on improving the quality of concrete by introducing admixture and low-dose nanomaterials instead of binder materials in concrete mixtures, achieving important results [30,31]. Li et al. [32] reported that proper nanomaterials can positively affect the workability and mechanical behaviors of nanomaterials reinforced concrete. Wang et al. [33] investigated the impact resistance of nanofiller modified concrete and found that nanofiller can significantly advance the impact toughness and mechanical behavior of concrete under a high-speed impact load.

The most commonly used nanoparticles in engineering primarily involve nano-SiO_2_, nano-CaCO_3_, nano-Al_2_O_3_, carbon nanofibers and so on [34,35,36]. Compared to other nanoparticles, nano-SiO_2_ (NS) has attracted more attention due to its larger specific surface area and higher reactivity. The volcanic ash reaction of NS can accelerate the dissolution of tricalcium silicate to form hydrated calcium silicate [37]. Several researchers have found that NS particles can improve the mechanical performance and durability of concrete [38,39]. Liu et al. and Mokhtar et al. [40,41] investigated that the addition of NS particles had the best effect in improving the mechanical performances of concrete. However, when NS content was greater than 2%, NS became self-dry and aggregate, resulting in strength losses and microcracks of cementitious composites. Obviously, the right number of NS particles can expressively improve the mechanical behaviors of concrete. Besides, many academics all over the world have conducted much research on NS–GPC in recent years [42,43,44,45]. Previous studies demonstrated that NS particles in FA-based geopolymer can improve the polymerization reaction degree of geopolymer, enhance the density of matrix and advance the mechanical properties of geopolymer through a chemical reaction, a filling effect and a crystal nucleus effect [46]. Moreover, NS has a low cost and excellent properties such as high elastic modulus and excellent acid and alkali resistance. Therefore, NS is a more suitable nanomaterial for crack resistance and toughness for GPC.

With the scale of hydraulic structures and marine structures in coastal areas becoming larger and more complex, the service environment of marine structures is also becoming more severe. Generally, the phenomena of high humidity, high temperature and high chloride concentration exist in many coastal areas. The strength and durability of many structures are seriously threatened under the coupling effect of a wet–thermal and chloride salt environment. There are various corrosive substances in the marine environment such as chloride ion, magnesium ion and sulfate radicals. Chloride ion erosion is the main reason for the durability damage of structures [47,48,49]. Chloride ions can penetrate into the concrete and react with C-A-H to form new salts, with which it is easy to produce large volume expansion and internal stress in the concrete, causing a dramatic decline of concrete strength [50,51]. Due to the cracks caused by volume expansion, more chloride ions will enter the members, which accelerates the speed of chloride ions infiltrating into the concrete and increases the corrosion of steel bars in the member, resulting in a decline of the durability of concrete structures [52,53]. Additionally, an environment of high humidity and high temperature will accelerate the diffusion speed of harmful ions in concrete and increase the corrosion process [54]. Consequently, good mechanical properties are important for preventing the excessive development of cracks, for delaying the diffusion of chloride ions into the members and improving the resistance of concrete to harmful ion corrosion. Due to the long-term effect of a coupling environment, the strength and durability of traditional concrete structures will be reduced, and the service life and safety will be threatened; hence, the performance of GPC under the effect of a coupling environment has attracted more attention. Çevik et al. [55] found that NS particle addition to FA-based GPC had the lower porosity in the internal structure of the matrix and enhanced its density, thus exhibiting better mechanical properties exposed to acid and salt solutions compared to the specimens without NS particles. Nuaklong et al. [56] found that the chloride penetration depth for recycled aggregate GPC was increased expressively with the addition of NS particles, and their results showed that NS-GPC had better resistance to chloride erosion compared to GPC without NS during long-term exposure.

For the concrete structures served under the coupling effect of a wet–thermal and chloride salt environment, the mechanical properties of compressive strength, tensile strength, elastic modulus and impact resistance are significant enough to ensure the safety of the structures. Specifically, the strength and elastic modulus of the concrete must meet the requirements of the bearing capacity and deformation resistance of structures. However, there are still few studies on GPC in complex conditions, especially the coupling effect of a wet–thermal and chloride salt environment. Therefore, in this study, the effect of NS content on the mechanical properties of GPC under the coupling effect of a wet–thermal and chloride salt environment were investigated via a number of tests, and the corresponding influence law and the corresponding mechanism were revealed. Based on several researchers, the FA–MK combination, as an aluminosilicate precursor for the synthesis of a geopolymer, results in GPC possessing excellent mechanical behaviors [11,12,13,14,15]. The expected results of this study are important for developing and enriching the basic theory of GPC and promoting the application of NS–GPC in hydraulic structure engineering and marine structure engineering.

## 2. Experimental Investigation

### 2.1. Materials

**Figure 1:**
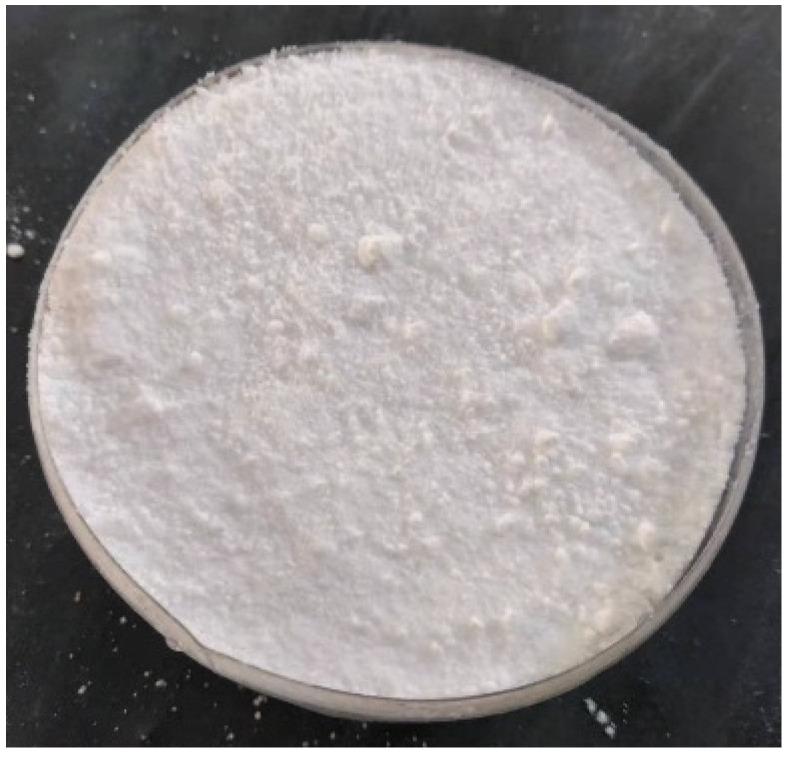
The appearance of powdery NS.

In this work, MK and FA were chosen as the geopolymer precursors, and the chemical compositions and physical properties of the MK and FA are presented in Table 1, Table 2 and Table 3, respectively. Sodium hydroxide, with 99% purity and sodium silicate, with 3.2 of the initial modulus, 1.41 of special gravity and 40% of solid content were used as alkaline activators. River sand with a fineness of 2.8 was used as a fine aggregate. Gravel with particle sizes of 5 to 20 mm was used as a coarse aggregate. A polycarboxylate superplasticizer with a 25% water reducing rate was used in this experiment. The main properties and appearance of NS are shown in Table 4 and Figure 1.

### 2.2. Mix Proportions and Specimen Preparation

In this experiment, the mix proportion of NS-GPC was based on the Chinese Standard (JGJ 55-2011) [57]. The water–binder ratio, the aggregate–binder ratio and the sand–aggregate ratio by weight were fixed as 0.52, 3.0 and 0.35, respectively. The dosage ratio of MK to FA was 3:2. After many attempts, the modulus of the alkaline activator was set to 1.3. To illustrate the influence of NS content on the mechanical properties of GPC under the coupling environment of wet–thermal and chloride salt, the NS fractions were chosen to be 0%, 0.5%, 1.0%, 1.5% and 2.0%. The mixed proportions of this experiment are summarized in Table 5, in which C represents the control group without NS under the natural environment, while N represents the test groups with different NS contents.

The uniform dispersion of NS is critical to obtaining concrete with good mechanical properties [58]. Firstly, the superplasticizer and NS were added to the alkali activator and stirred at high speed, accelerating the dispersion of NS. Then, river sand, FA and MK were mixed and stirred for about 2 min. Next, a prepared solution of alkali activator, NS and superplasticizer were poured into the mixture and stirred for 2 min. Afterwards, gravel was added to the mixture and stirred for about 2 min. All samples were demolded 24 h later in an ambient temperature environment with a relative humidity of 40–60% and curing for 28 days (20 °C and 95% relative humidity). Eventually, all samples were put in the environmental simulation test chamber for 60 days before the testing of mechanical properties.

### 2.3. Simulation on Coupling Effect of Wet–Thermal and Chloride Salt Environment

**Figure 2:**
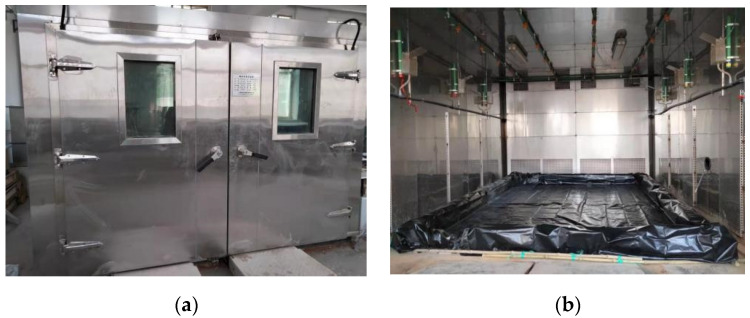
The environmental simulation test chamber. (**a**) Overall appearance; (**b**) Internal structure.

To investigate the mechanical properties of NS-GPC under the coupling effect of a wet–thermal and chloride salt environment, the effective way is to simulate the environment with high humidity, high temperature and salt content in the environment simulation test chamber, and then place the specimens in the test chamber to conduct the degradation test. By increasing the humidity, the temperature and the salt content, the effect of a wet–thermal and chloride salt coupling environment on NS–GPC was enhanced, and the accelerated test was then achieved. The overall appearance and internal structure of the environmental simulation test chamber are shown in Figure 2a,b.

Water is the carrier for the diffusion of chloride ions in NS–GPC, and therefore the influence of environmental humidity on the diffusion of chloride ions should be considered. The diffusion velocity of chloride ions will decrease and the effect of the acceleration test will not be achieved if the relative humidity in NS–GPC is too low. Furthermore, the average relative humidity in coastal cities in July is between 76% and 89% [59]. Hence, the relative humidity needs to be further improved; the relative humidity was set to 100% to achieve the acceleration test.

The environment temperature is important for the diffusion of chloride ions in a solution. As the temperature grows, the solubility of inorganic salts will increase, and the water molecules will become more active with the increased diffusion velocity of chloride ions. Therefore, it is necessary to raise the test temperature appropriately. In this experiment, the calculation of the optimal test temperature was carried out according to the relationship between the diffusion coefficient of chloride ions and temperature given by Nernst–Einstein in order to accelerate the test [60]. Finally, the temperature in the environmental simulation test chamber was determined to be 45 °C.

According to the Chinese Standard (GB/T 2423.17-2008) [61] and other situations of chloride ion environment simulation tests [62], 5% sodium chloride (NaCl) solution was used in this experiment. The NaCl solution was prepared using the solid NaCl with a purity of 99.7% and distilled water. All specimens were immersed in the prepared salt solution, and the salt–fog environment in the test chamber were kept at a constant humidity and salt concentration in the simulation test to achieve the indoor accelerated erosion effect expected in this experiment.

Referring to the current environmental coupling simulation test [63] and combining the test objective of this study and the performance of the environmental simulation test chamber, the relative humidity, temperature and the concentration of the salt solution were set to 100%, 45 °C and 5%, respectively. Furthermore, the wet–dry cycle acceleration method was adopted, and the test duration was determined to be 60 days, including 10 test cycles. To achieve the effect of indoor accelerated erosion, all specimens were immersed for 3 days and air-dried for 3 days in each wet–dry cycle. Besides, the method of spraying and soaking of the salt solution was utilized to maintain a constant relative humidity and salt concentration.

### 2.4. Cube Compressive and Splitting Tensile Tests

**Figure 3:**
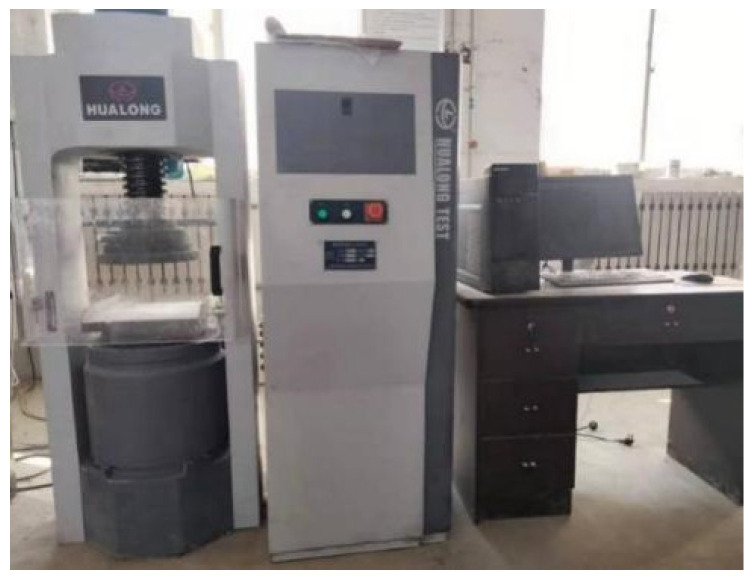
The testing apparatus of the cube compressive and splitting tensile test.

The cube compressive and splitting tensile strength of NS–GPC were measured following the Chinese Standard (GB/T50081-2019) [64]. Three 100 × 100 × 100 mm^3^ cube specimens from each mixture were cast. The tests were performed using an electron-hydraulic servo pressure testing device with a maximum range of 2000 kN. The loading rates for cube compressive and splitting tensile tests were set at 0.5 MPa/s and 0.07 MPa/s, respectively. The test device is shown in Figure 3. The final results were the average strength of the three specimens.

### 2.5. Elastic Modulus Test

Referring to the Chinese Standard (GB/T50081-2019) [64], the elastic modulus test of NS–GPC was conducted in this study. An electron-hydraulic servo testing machine with a loading rate of 0.5 MPa/s was used. A set of three 100 × 100 × 300 mm^3^ prisms were prepared for each mix proportion of the axial compressive and elastic modulus test to acquire accurate values. The average value of the three prisms was taken as the result. According to the recent test [28], the compressive elastic modulus can be calculated as follows:

(1) $$E_{c}=\frac{F_{a}-F_{0}}{A}\times \frac{L}{\Delta n}$$

where, *E_c_* is elastic modulus (MPa); *F_a_* is the load under one-third of axial compressive strength (N); *F*_0_ is the beginning load under 0.5 MPa (N); *A* is the bearing area (mm^2^); *L* is the measuring range (mm); and Δ*n* is the mean value of deformation on both sides of the prism at the last loading from *F*_0_ to *F_a_* (mm).

### 2.6. Impact Resistance Test

**Figure 4:**
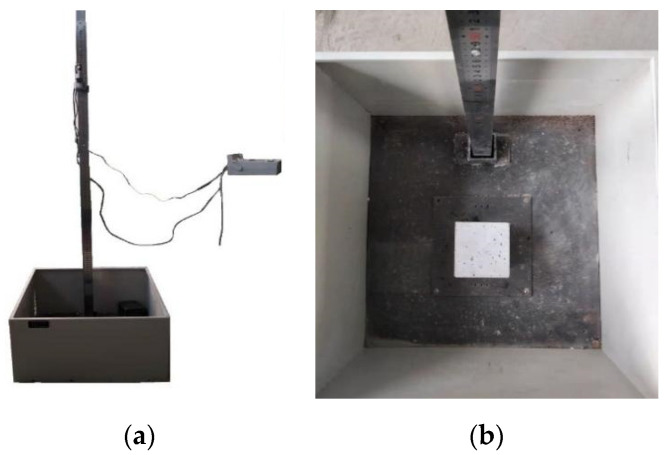
The testing apparatus for an impact resistance test. (**a**) Overall appearance; (**b**) Internal structure.

The impact resistance test of NS–GPC was performed on a drop hammer test machine. Figure 4a,b present the overall appearance and internal structure of the testing apparatus in an impact resistance test. Five 100 × 100 × 100 mm^3^ cube specimens were prepared for each group. First, the sample was put in the testing device, and then the hammer with an elevation of 1000 mm was dropped. One impact procedure was set as a cycle. The cracking and failure on the surfaces of specimens were recorded after each impact. Impact numbers of initial crack and ultimate failure in the specimen were set as N_1_ and N_2_, respectively. The difference value of N_2_-N_1_ was used to describe the impact toughness of NS–GPC. A total of five specimens were tested in each working condition, the maximum and minimum values were eliminated and the average value was used as the final result.

### 2.7. Microscopic Test

The microstructure of NS–GPC under the coupling effect of a wet–thermal and chloride salt environment was investigated using a KYKY-EM6200 scanning electron microscope (SEM). After the strength determination of each specimen, pieces of less than 1 cm^3^ were dried in a furnace at 60 °C and sprayed with gold before the test. The development and phase changes of the microcracks were observed by SEM at various magnifications.

## 3. Results and Discussion

### 3.1. Cube Compressive Strength

**Figure 5:**
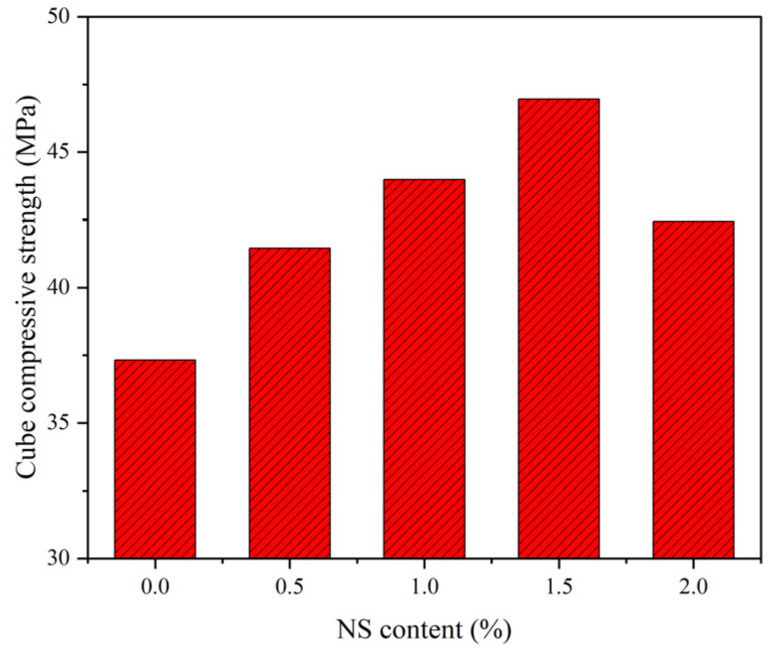
The cube compressive strength of NS–GPC.

Cube compressive strength is the most commonly used compressive strength to evaluate the compressive properties of concrete. According to the related results, the addition of NS can improve the compressive strength of traditional cement concrete and geopolymer composites through the effect of the secondary hydration and the effect of filling NS particles [11,31]. However, the effect of NS particles on GPC under the coupling effect of a wet–thermal and chloride salt environment is unknown. Figure 5 exhibits the influence of NS content on the cube compressive strength of NS–GPC under the coupling effect of a wet–thermal and chloride salt environment. Experimental results revealed that the cube compressive strength of test groups with NS were expressively enhanced compared to the control group without NS. Obviously, the cube compressive strength of NS–GPC exhibited a developmental tendency of increasing primarily and decreasing later as the growth of NS content. Specifically, when the NS content was 1.5%, the cube compressive strength achieved an optimal value of 46.95 MPa, which was a rise of 25.8% compared to that of the control group. It can be concluded that 1.5% is an optimal NS content for an improvement in the cube compressive strength of GPC.

**Figure 6:**
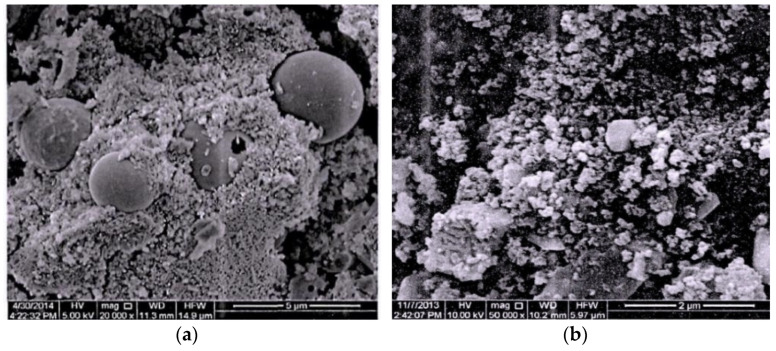
The microstructure of the geopolymer. (**a**) Group C; (**b**) Group N-1.5.

The major reasons for the improved properties of concrete are the nano-filling effect and pozzolanic reaction of NS particles [65]. Besides, it can be due to the pozzolanic activity of nano-SiO_2_, which leads to more aluminosilicate gels in mortar as a complement to the Si-O bonds [45]. Through an SEM test, the microstructures of geopolymer are presented in Figure 6a,b. As mentioned previously, the appropriate dosage of NS particles can effectively fill micro-pores in concrete to improve the distribution of pores and also promote the formation of the geopolymer cementation system to make the structure of concrete more uniform, compact and orderly, which can then improve the compression performance of concrete. However, too large content an NS particle content may have an adverse influence on the compressiveness of GPC. The reason was illustrated to be that the water requirement increased due to the second hydration of NS particles. The excessive NS particles aggregate together and become the defect inside the concrete composites [66,67]. Therefore, the cube compressive strength of GPC with an addition of 2% NS was lower than that of the GPC with an addition of 1.5% NS.

### 3.2. Splitting Tensile Strength

**Figure 7:**
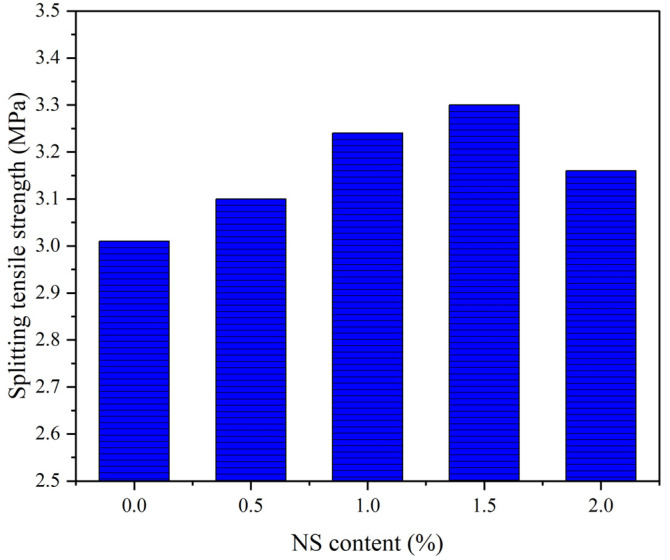
The splitting tensile strength of NS–GPC.

For the concrete structures under the coupling effect of a wet–thermal and chloride salt environment, many concrete components will be subjected to the action of tensile stress. The direct tensile test is the best method to evaluate tensile properties of concrete composites. However, it is very difficult to carry out a test of direct tensile due to the high requirements needed of the test apparatus. As a result, the splitting tensile strength test is often applied. Figure 7 depicts the influence of the NS content on the splitting tensile strength of NS–GPC under the coupling effect of a wet–thermal and chloride salt environment. Test results indicated that the splitting tensile strength was increased by adding NS particles with the content from 0.5% to 1.5%, reduced, however, with higher fractions. Distinctly, the splitting tensile strength of NS–GPC reached an optimal value of 3.30 MPa, which was an enhancement of 9.6% compared to that of the control group without NS. It can be concluded that 1.5% is an optimal NS content for an improvement in the splitting tensile strength of GPC.

**Figure 8:**
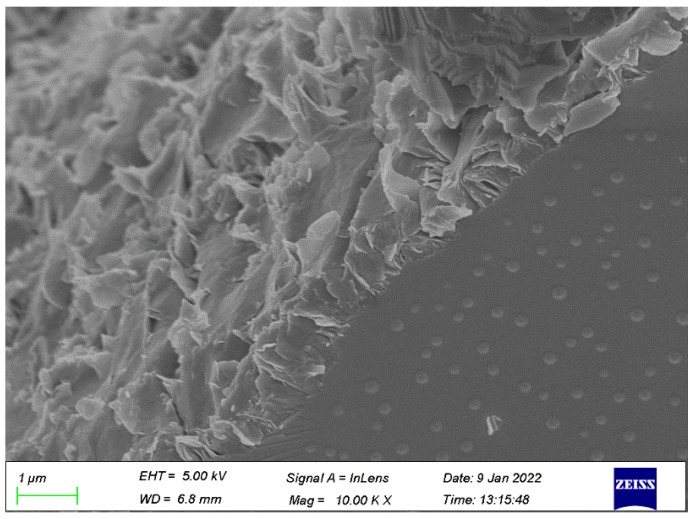
The ITZ of NS–GPC.

As mentioned previously, growth in the splitting tensile strength of GPC was caused by NS resulting from the mechanisms of the seeding effect, filling effect, and chemical reactions with FA [68]. Moreover, the interfacial transition zone (ITZ) is the weak part of the concrete, which seriously affects the concrete performance [69]. As shown in Figure 8, the improvement of the ITZ of GPC by NS greatly enhanced the density of GPC. The appropriate content of NS particles improved the splitting tensile strength obviously. The improvement in splitting tensile strength was attributed to the small size of the NS particles, which increased the compactness of GPC by filling the pores of the matrix. For the cement concrete, the same results could be found, which indicated that the addition of NS greatly improved the splitting tensile strength of the geopolymer composites [45]. However, excessive NS particles were difficult to disperse uniformly in the GPC mixture, which affected the stability of the three-dimensional network structure of GPC. As a result, the splitting tensile strength of GPC, with an addition of 2% NS, was lower than that of the GPC with an addition of 1.5% NS.

### 3.3. Elastic Modulus

**Figure 9:**
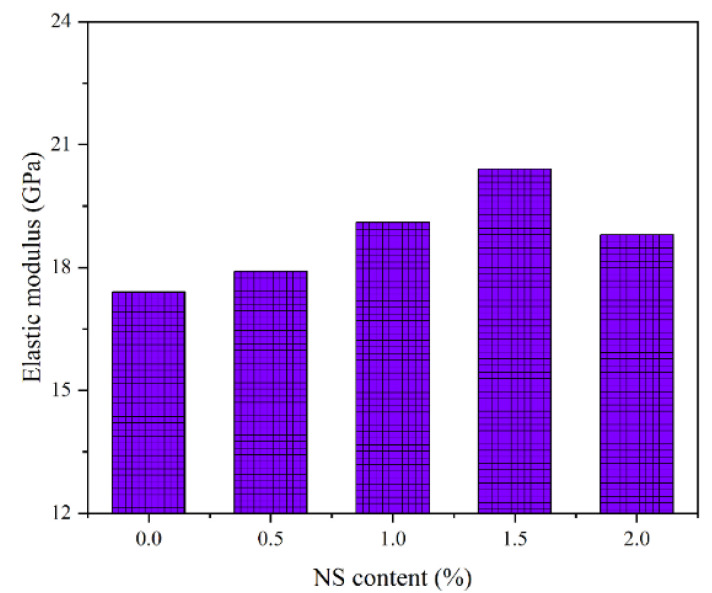
The elastic modulus of NS–GPC.

For the traditional cement concrete materials, elastic modulus is usually used to evaluate the deformation resistance of the concrete. The effect of addition of admixtures on elastic modulus depends on the effect on compressive strength. The addition of NS particles often increases the elastic modulus of cement concrete based on the related results []. Since the elastic modulus is so important for the deformation resistance of the concrete, the influence of NS on elastic modulus of GPC under the coupling effect of a wet–thermal and chloride salt environment should be discussed. Figure 9 depicts the elastic modulus of NS-GPC under the coupling effect of a wet–thermal and chloride salt environment with different NS contents. Obviously, the elastic modulus of GPC exhibited a tendency of increasing primarily and decreasing later as the increment of NS content. In general, NS enhanced the elastic modulus of GPC. When the NS content was 1.5%, the elastic modulus of NS-GPC reached the maximum value of 20.4 GPa, which was an increase of 17.2% compared with that of control group without NS. It can be concluded that 1.5% is an optimal NS content for the improvement in elastic modulus of GPC. NS particles can promote the formation of network structure in the geopolymer cementation system, and improve the integrity of concrete, so as to increase the elastic modulus of GPC. This is because the NS dissolved and hydrated, yielding more (N, C)-A-S-H gel, thus increasing the deformation resistance of the matrix [14]. Nevertheless, too much of NS will consume excessive free water in concrete and be assembled together in the geopolymer matrix, which inhibited the polymerization reaction, which adversely affected the stability of the network structure at the molecular level, resulting in the decrease of elastic modulus of GPC. Therefore, the elastic modulus of GPC with an addition of 2% NS was lower than that of the GPC with an addition of 1.5% NS.

### 3.4. Impact Resistance

**Figure 10:**
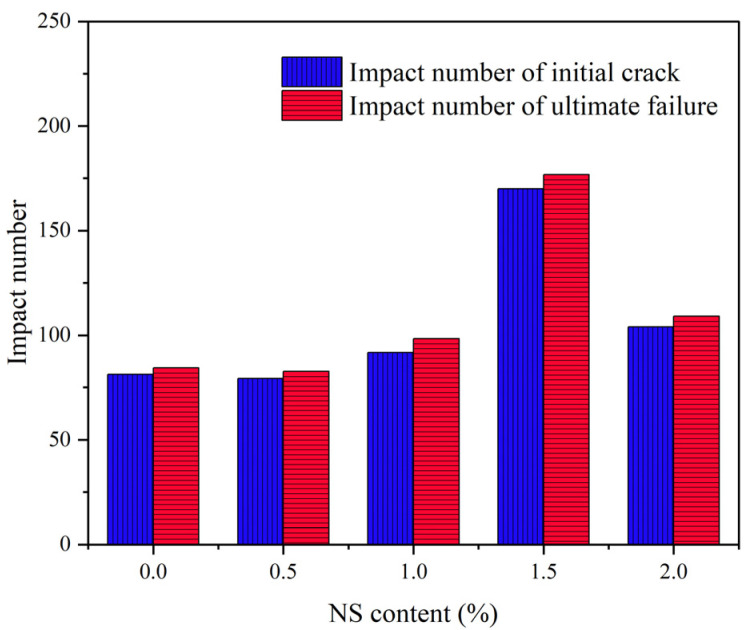
The influence of NS content on impact numbers.

**Figure 11:**
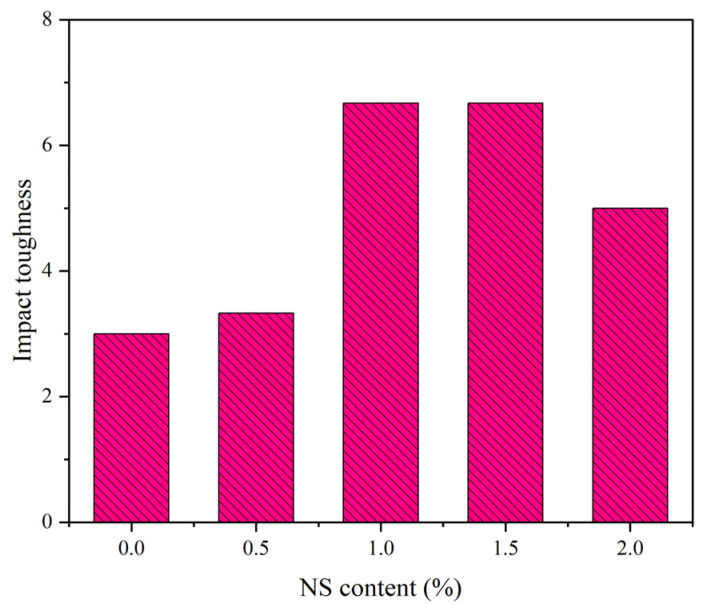
The impact toughness of NS–GPC.

Figure 10 and Figure 11 exhibit the influence of NS on impact toughness, impact numbers of initial crack and the ultimate failure of NS–GPC under the coupling effect of a wet–thermal and chloride salt environment. Experimental results indicated that impact toughness, impact numbers of initial crack and the ultimate failure of NS–GPC increased because of an appropriate dosage of added NS. When NS content was less than 1.5%, impact toughness, impact numbers of initial crack and the ultimate failure enhanced with the rise of NS content. Specifically, when NS content was 1.5%, impact toughness, impact numbers of initial crack and the ultimate failure reached maximums of 6.67, 170 and 176, respectively, which was an increase of 122.3%, 109% and 109.5% compared to that of the control group without NS. When NS content was greater than 1.5%, impact toughness, impact numbers of initial crack and the ultimate failure declined. It can be concluded that the impact resistance of GPC with NS is indeed better than that of GPC without NS.

**Figure 12:**
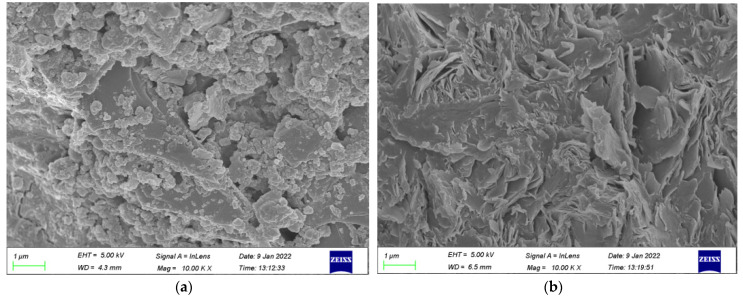
The microstructure of the geopolymer. (**a**) Group C; (**b**) Group N-1.5.

Figure 12a,b exhibits the SEM images of group C and group N-1.5, respectively. Results revealed that the incorporation of NS can effectively fulfill the pores and tiny cracks in GPC and make the internal structure of concrete more compact so that more of the impact energy can be absorbed by GPC materials. Additionally, the small size effect of NS can advance the impact resistance of GPC. However, excessive NS will weaken the reinforcement influence of NS on impact resistance of GPC due to an inhibiting of the formation of internal three-dimension stable structure, which is why an addition of 2% NS lowers the impact toughness, impact numbers of initial crack and the ultimate failure of GPC compared to 1.5%. [70].

### 3.5. Coupling Effect of Wet–Thermal and Chloride Salt Environment

**Figure 13:**
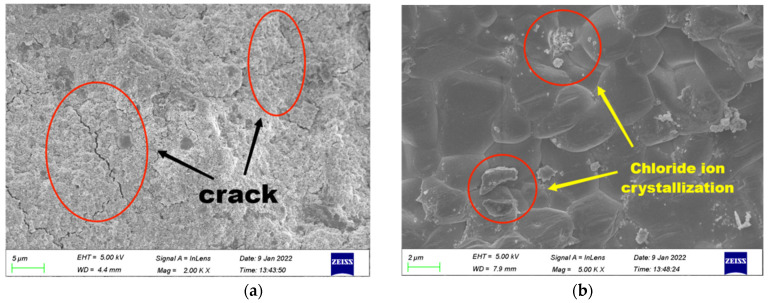
The microstructure of NS–GPC under a coupling environment. (**a**) Microscopic cracks; (**b**) Chloride ion crystallization.

According to the erosion mechanism of inorganic salt on concrete and the weathering mechanism of geopolymer [61], chloride ions will first enter the surface of GPC through the action of diffusion, adsorption, infiltration and convection under the transportation of water. Then, they are transformed into expansive corrosion products via chemical reaction or solidified in a transition zone via physical crystallization, which itself caused the further development for the original cracks and pores on the surface area of GPC, after which more new cracks formed, as exhibited in Figure 13a,b. With the accumulation of erosion action, the surface defects of GPC increased continuously, resulting in spalling on the surface area of GPC. The chloride ions then continued to erode the new surface layer from the outside to the inside with a large number of cycles until GPC was completely destroyed.

**Figure 14:**
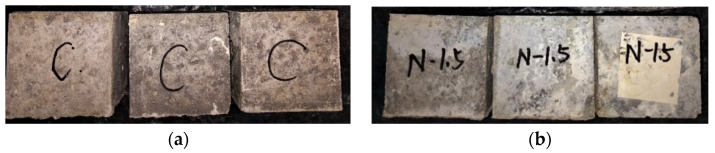
The appearance changes of the GPC specimen after the wet–dry cycle. (**a**) Samples of group C; (**b**) Samples of group N-1.5.

In this experiment, the environment with high humidity, high temperature and salt content accelerated the physical corrosion action of chloride ions and water on GPC. Figure 14a,b exhibit the appearance changes of the GPC specimen after the wet–dry cycle. Obviously, the crystallization of white salt particles accumulated on the outer surface of the specimens, and the crystallization phenomenon was more obvious in the rough surface with enormous pores. Additionally, the outer surface of the specimen not subjected to wet–dry cycles was bright gray, while the outer surface of the specimen subjected to several cycles of wet–dry was dark gray, and obvious microscopic cracks and chloride ion crystallization could be observed for both specimens.

**Figure 15:**
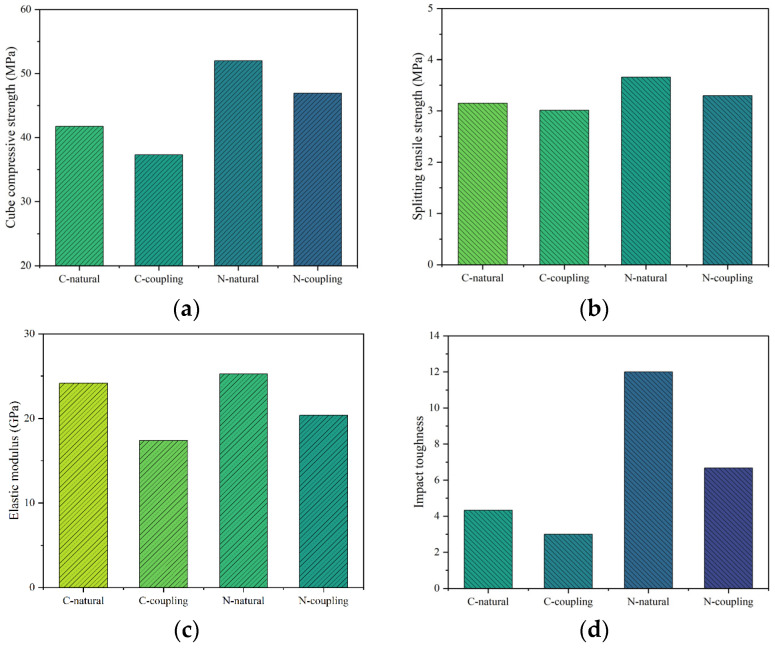
The mechanical properties of GPC under different environments. (**a**) Cube compressive strength; (**b**) Splitting tensile strength; (**c**) Elastic modulus; (**d**) Impact toughness.

In this study, the changes in mechanical properties of GPC under the coupling environment of wet–thermal and chloride salt are presented in Figure 15. The control group under the natural environment was denoted as C-natural. The control group under the coupling environment of wet–thermal and chloride salt was denoted as C-coupling. The test group mixed with 1.5% NS content under the natural environment denoted as N-natural. The test group mixed with 1.5% NS under the coupling environment of wet–thermal and chloride salt was denoted as N-coupling. From the figures, it can be found that the changes of mechanical properties of GPC under the coupling environment of wet–thermal and chloride salt are lower than those under the natural environment. The cube compressive and splitting tensile strength, elastic modulus and impact toughness of GPC without NS under the coupling environment of wet–thermal and chloride salt were reduced by 10.6%, 4.4%, 30.0%, and 30.7%, separately compared with that of control group under the natural environment. When NS content was 1.5%, the cube compressive and splitting tensile strength, elastic modulus and impact toughness of GPC under the coupling environment of wet–thermal and chloride salt were decreased by 9.7%, 9.8%, 19.2% and 44.4%, respectively, relative to that of the GPC under the natural environment. Test results indicated that NS had an inhibitory influence on the decline of the elastic modulus and impact toughness of GPC; however, NS contents had almost no obvious influence on the decline of the cube compressive and splitting tensile strength of GPC. In general, it can be concluded that NS can actively resist the severe environmental impact on GPC.

## 4. Conclusions

In this study, simulation tests of the coupling effect of a wet–thermal and chloride salt environment were carried out. The effect of NS content on the mechanical behaviors—including cube compressive and splitting tensile strength, elastic modulus and the impact resistance of GPC after being subjected to the coupling effect of a wet–thermal and chloride salt environment—was investigated. The primary conclusions can be summarized as follows:

(1) NS can effectively enhance the cube compressive strength of GPC under the coupling effect of a wet–thermal and chloride salt environment. The cube compressive strength of NS–GPC exhibited a developmental tendency to increase primarily and decrease later as the growth of NS substitute rate. Specifically, when the NS content was 1.5%, the cube compressive strength achieved an optimal value of 46.95 MPa, which was a rise of 25.8% compared to that of the control group without NS.
(2) The addition of NS improved the splitting tensile strength of GPC under the coupling effect of a wet–thermal and chloride salt environment. When NS content increased from 0.5% to 2.0%, the splitting tensile strength of NS–GPC under the coupling effect of a wet–thermal and chloride salt environment presented a changing law of increasing primarily and decreasing later as the NS content increased. Distinctly, the splitting tensile strength of NS–GPC reached an optimal value of 3.30 MPa, which was an enhancement of 9.6% compared to that of the control group without NS.
(3) It can be concluded through experiments that 1.5% is an optimal NS content for reinforcement in the elastic modulus of GPC under the coupling effect of a wet–thermal and chloride salt environment. Additionally, the elastic modulus of GPC exhibited a tendency of increasing primarily and decreasing later as the rise of NS content. An excess of NS will adversely affect the stability of the network structure at the molecular level, resulting in a decrease of the elastic modulus of GPC.
(4) NS distinctly improved the impact resistance of GPC under the coupling effect of a wet–thermal and chloride salt environment. When the NS content was increased from 0.5% to 2.0%, impact toughness, impact numbers of initial crack and ultimate failure of GPC under the coupling effect of a wet–thermal and chloride salt environment exhibited a tendency of increasing primarily and declining later. Moreover, the SEM test results revealed that the incorporation of NS particles can effectively fulfill the pores and tiny cracks in GPC and make the internal structure of concrete more compact, allowing for more of the impact energy to be absorbed by GPC materials.
(5) Compared to the control group in the natural environment, the mechanical properties of NS–GPC decreased under the coupling effect of a wet–thermal and chloride salt environment. Experimental results indicated that NS had an inhibitory influence on the decline of the elastic modulus and the impact toughness of GPC; however, NS contents had almost no obvious influence on the decline of the cube compressive and splitting tensile strength of GPC. In general, it can be concluded that NS can actively resist the severe environmental impact on GPC.

## Figures and Tables

**Table 1:** The chemical compositions of MK and FA.

Chemical Compositions (wt.%)	SiO_2_	Al_2_O_3_	Fe_2_O_3_	CaO + MgO	K_2_O + Na_2_O	SO_3_
MK	54	43	≤1.3	≤0.8	≤0.7	–
FA	60.98	24.47	6.70	5.58	–	0.27

**Table 2:** The physical properties of MK.

Whiteness (%)	Activity Index (%)	Availability of Lime (mL)	Average Particle Size (µm)	Ignition Loss (%)
75	12	1350	1.2	0.5

**Table 3:** The physical properties of FA.

Water Demand Ratio (%)	Standard Consistency (%)	Bulk Density (g/cm^3^)	Density (g/cm^3^)
105	47.1	0.77	2.16

**Table 4:** The physical properties of NS.

SiO_2_ Content (%)	Specific Surface Area (m^2^/g)	Bulk Density (g/cm^3^)	Average Particle Size (nm)	pH
99.5	200	0.035	30	6

**Table 5:** The mix proportions of NS–GPC.

Mix ID	FA	MK	NS	Alkali Activator	River Sand	Coarse Aggregate	Water-Reducing Agent
	kg/m^3^	kg/m^3^	%	kg/m^3^	kg/m^3^	kg/m^3^	%
C-0	195.0	273.0	0	418.5	577.4	1072.4	2.5
N-0.5	194.0	271.5	0.5	418.5	577.4	1072.4	2.5
N-1.0	193.0	270.0	1.0	418.5	577.4	1072.4	2.5
N-1.5	192.0	269.0	1.5	418.5	577.4	1072.4	2.5
N-2.0	191.0	267.6	2.0	418.5	577.4	1072.4	2.5
